# Understanding the dynamics of notification and implementation of Article 5.3 across India’s states and union territories

**DOI:** 10.1136/tobaccocontrol-2021-057119

**Published:** 2022-02-09

**Authors:** Shalini Bassi, Rob Ralston, Monika Arora, Aastha Chugh, Gaurang P Nazar, Jeff Collin

**Affiliations:** 1 HRIDAY, Delhi, India; 2 Global Health Policy Unit, School of Social and Political Science, The University of Edinburgh, Edinburgh, UK; 3 Global Health Policy Unit, The University of Edinburgh, Edinburgh, UK

**Keywords:** tobacco industry, public policy, low/middle income country

## Abstract

**Introduction:**

In federal systems, state and local governments may offer opportunities for innovation in implementing the WHO Framework Convention on Tobacco Control (FCTC). This paper explores the implementation of WHO FCTC Article 5.3 within India’s federal system, examining how its guidelines have been operationalised across states and union territories.

**Methods:**

Interviews with officials from government and civil society organisations across key states, and a document review of state government and district administration notifications adopting Article 5.3 guidelines between 2015 and 2019.

**Results:**

The data reveal subnational leadership in formulating intersectoral committees, which are designed to limit interactions with the tobacco industry, and corresponding measures to reject partnership and conflicts of interest for government officials. There are notable omissions across states and union territories in adoption of key Article 5.3 guidelines; only four districts and state governments refer to regulating aspects of ‘socially responsible’ industry activities, and no notifications include measures to prevent the tobacco industry receiving preferential treatment or requiring that information provided by industry actors be transparent and accountable. Interview data indicate that dynamics of notification across states have been shaped by lesson drawing and the catalytic role of civil society. The adoption of protocols is impacting on the practices of health officials, but there are concerns about engagement by other departments and the regulatory capacity of empowered committees.

**Conclusion:**

The spread of state- and district-level policies illustrates opportunities federal structures can provide for accelerating tobacco control. Given significant omissions and policy tensions, there remains a need for national action to build on these innovations, including in revisions to India’s tobacco control legislation.

## Introduction

While implementation of the WHO Framework Convention on Tobacco Control (FCTC) is widely seen as requiring a whole-of-government approach,^1^ there has been limited empirical research of coordination in multilevel political systems.^2 3^ Investigation of coordination mechanisms for tobacco control has focused more on horizontal interaction across government sectors^4 5^ than vertical relationships between different levels of government. Alongside a primary focus on the national level in considering how member states have implemented international obligations under the FCTC,^6 7^ this has led to a comparative neglect of subnational dynamics in studies of FCTC implementation. This is despite the potential strategic advantages to tobacco control of understanding the distribution of responsibilities across different levels of government,^8^ of scope to adopt stronger policies at local or state level than might be attainable nationally^9 10^ and of dynamics of policy learning or transfer whereby subnational innovation can catalyse subsequent national approaches.^2 11^

The need for coordinated, whole-of-government approaches to tobacco control policy is recognised in the FCTC text, which requires parties to implement multisectoral tobacco control strategies.^12^ The treaty also embodies a distinctive model of health governance in Article 5.3^13^ that addresses the threat of tobacco industry interference, obligating parties to protect public health policy making from the commercial and vested interests of industry actors. Improved implementation of Article 5.3 was identified by the WHO FCTC Impact Assessment Expert Group as the ‘single highest priority’ in advancing the treaty and strengthening its impact.^6^

As a general obligation, Article 5.3 is applicable to all government departments, and the WHO guidelines recognise that minimising industry interference necessitates implementation across multiple levels of government; its provisions apply to government officials ‘of any national, state, provincial, municipal, local or other public or semi/quasi-public institution.’^14^ This need for a multisectoral and multilevel approach to tobacco control governance has not received significant attention in the existing literature on Article 5.3.

This paper focuses on India as a key context within which to explore the multilevel dynamics of Article 5.3, analysing the notification of guidelines to implement its provisions across India’s states and union territories (with Jammu and Kashmir classified as a Union Territory following the Jammu and Kashmir Reorganisation (Amendment) Bill, 2021). As a federal political system with a population of 1.38 billion, India represents a significant case study for understanding multilevel FCTC implementation. Indian federalism entails the distribution and decentralisation of responsibility from central government to state governments, in which the Constitution of India specifies legislative and policymaking functions across different levels of government.^15 16^ Central and state governments share legislative and administrative responsibility for welfare policies,^17^ notably education, pensions and social security.^18^ Public health is distinctive in the autonomy delegated to states under the constitution,^18 19^ creating the potential for subnational policy innovation and patterns of divergence and convergence across individual states.

Tobacco control reflects this division of responsibilities between central government and states.^20 21^ At national level, FCTC measures have been integrated into India’s primary tobacco control law, the 2003 Cigarettes and Other Tobacco Products Act (COTPA). COTPA confers responsibility on the central government to ‘make rules to carry out the provisions of this Act’^22^ including for pictorial health warnings, smoke-free public places and advertising, promotion and sponsorship restrictions, while states are charged with enacting and implementing measures at subnational and district levels.^23^ The autonomy enjoyed by states in tobacco control governance has provided opportunities for policy innovation in issues that are not included in COTPA. Arguably COTPA’s most significant omission is the absence of measures to prevent tobacco industry interference (despite a broad declaration that ‘it is expedient in the public interest that the Union should take under its control the tobacco industry’).^22^ In seeking to address this regulatory gap, state and district governments have issued notifications and developed protocols to implement Article 5.3, amid concerns about tobacco industry interference and its engagement with government policy programmes at national and state levels.^24 25^

This paper assesses the scope of policies adopted across the 11 states, 1 union territory and in 2 districts that have notified Article 5.3 measures in the period 2015–2020, comparing such measures with eight recommendations provided in FCTC Article 5.3 implementation guidelines.^14^ The paper seeks to explore the dynamics of policy innovation in implementing Article 5.3 across multiple levels of government, to consider how policies and practices are impacting on the work of state officials, and to analyse barriers and facilitators to more effective implementation in a federal system.

## Methods

This research draws on two sources of data: interviews and document analysis. The first source was an analysis of gazette prints of notifications accessed via state and district government websites and ICMR National Institute of Cancer Prevention and Research archive of Article 5.3 notifications.^26^ These documents varied in structure and length (between 1 and 7 pages), with several jurisdictions including circulars, codes of conduct and administrative protocols. The documents were assessed against the eight core recommendations in the WHO guidelines for the implementation of Article 5.3.^14^ In addition, we assessed institutional mechanisms to implement Article 5.3 measures within notifications, specifically the presence or absence of intersectoral committees (empowered committees) and codes of conduct for government officials relating to interactions with the tobacco industry. MA formulated the idea of comparing state and district notifications against Article 5.3 recommendations, with documents coded by SB and second-coded by RR and JC. Interpretive differences were resolved in deliberation between the coders and consensus reached about revisions to the analysis.

To analyse how Article 5.3 has been developed and implemented, we conducted 26 in-depth, semi-structured interviews with officials and stakeholders across notifying states and union territory between January 2019 and October 2020 (table 1). These included interviews with officials within state health departments (n=10) and departments and agencies beyond health (n=5); public health advocates and stakeholders with experience of tobacco control governance and notification processes across different states (n=8); representatives of national-level civil society organisations (CSOs) (n=2) and a legal consultant (n=1). Interviewees were based in seven states: Bihar, Himachal Pradesh, Karnataka, Mizoram, Punjab, Tamil Nadu and West Bengal. Interviews that had been planned with officials in the other five states that have issued notifications proved unfeasible due to COVID-19 mitigation measures in India. These included travel restrictions and limited availability of officials who had taken on additional COVID-19-related policy responsibilities. A limitation of this research is, therefore, that interviews were conducted in most, but not all, state and union territories that have notified Article 5.3. This means that our findings are unable to present a comprehensive narrative of the dynamics and impacts of notification.

**Table 1:** Overview of interviewees

State	No of interviewees
Bihar	5
Himachal Pradesh	4
Karnataka	4
Mizoram	4
Punjab	4
Tamil Nadu	1
West Bengal	1
National level	3
Total	26

A semi-structured interview schedule covered three key themes: awareness of FCTC Article 5.3, dynamics of Article 5.3 adoption and notification across state and union territories and the extent of implementation of Article 5.3 guideline recommendations. The semi-structured approach enabled the interview schedule to be adapted to different contexts, in which interviewees were asked more specific questions relating to the Article 5.3 notification issued in their state or union territory.

MA, SB and AC developed an initial list of interviewees, based on publicly available information and in-depth knowledge of tobacco control policy at different levels of governance. Interviewee selection was also guided by ‘snowball’ sampling,^27^ using professional networks and recommendations made by other interviewees. Interviews varied in length between 15 and 95 min (with most between 30 and 40 min), with 23 interviews conducted in-person and 3 interviews using teleconferencing software.

Interviewees were asked to review and sign a consent form (or provide verbal consent in the case of interviews conducted via teleconferencing software) allowing interviews to be recorded and for data to be used in research publications. Interviews were conducted in English, transcribed and anonymised, with transcripts coded in NVivo 12 using a thematic framework developed via descriptive analysis, followed by conceptual coding of the interview data.^28^ The approach started with descriptive codes about notification processes and contextualised with reference to Article 5.3 and its provisions. This allowed iterative identification of themes through analysis and reanalysis of interview transcripts. In presenting the interview data, we differentiate between the individual states that interviewees worked in. This approach aims to balance clarity about institutional and geographic context, with the need to maintain the anonymity of interviewees.

Interviewees were approached by MA, with interviews conducted by GPN, SB and AC. Transcripts were coded by SB and RR, with input from JC and AC. Preliminary findings were reviewed at a Global Challenges Research Fund consortium meeting in Delhi, India, in January 2020 and developed via coordination calls between SB, RR, AC and JC. The research obtained ethical approval from the Indian Ministry of Health and Family Welfare Screening Committee, Centre for Chronic Disease Control and the University of Edinburgh.

## Results

### Variations in Article 5.3 measures across state and district notifications

A comparative analysis of measures within Article 5.3 notifications across the 11 states, 1 union territory, and 2 district government notifications included here highlights significant policy divergence (table 2). As one interviewee remarked, “in a country like India with so many states, and with health being a state subject, each state [can] do their own thing.” But there are also broad patterns in implementation with substantive convergence around key issues addressed and neglected, while Punjab’s 2015 document appears to have provided a template for subsequent notifications in other jurisdictions.

**Table 2:** Comparison of notifications across India’s state and union territories and WHO guidelines for implementation of Article 5.3

State	Punjab	Mizoram	West Bengal		Himachal Pradesh	Mahar ashtra	Bihar	Jammu and Kashmir	Tamil Nadu	Jharkhand	Karnataka	Kerala	Uttar Pradesh	Megh alaya
District			Darjeeling	Howrah										
Year	2015	2016	2016	2016	2017	2017	2017	2017	2017	2018	2019	2019	2019	2019
Article 5.3 recommendations														
Raise awareness about the addictive and harmful nature of tobacco products and about tobacco industry interference with parties’ tobacco control policies														
Establish measures to limit interactions with the tobacco industry and ensure the transparency of those interactions that occur														
Reject partnerships and non-binding or non-enforceable agreements with the tobacco industry														
Avoid conflicts of interest for government officials and employees														
Require that information provided by the tobacco industry be transparent and accurate														
Denormalise and, to the extent possible, regulate activities described as ‘socially responsible’ by the tobacco industry, including but not limited to activities described as ‘corporate social responsibility’														
Do not give preferential treatment to the tobacco industry														
Treat state-owned tobacco industry in the same way as any other tobacco industry														
**Mechanisms for implementation**														
Code of conduct														
Empowered committee														

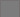
Broad consistency; 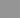
Limited provision; 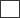
Omitted completely.

The establishment of an inter-departmental ‘empowered’ committee, to include representatives from key departments such as finance, agriculture, trade and commerce, is tasked with implementing measures to limit interactions with the tobacco industry; this has become a key mechanism adopted by two-thirds of notifying states. More broadly, most notifications are explicitly underpinned by the recognition of a fundamental conflict of interest between the tobacco industry and public health goals. For example, the Kerala government cites the need to protect tobacco control policies from ‘any kind of interference from the tobacco industry’ as the rationale for establishing an empowered committee, while the Mizoram protocol notes that it has been developed ‘due to the increasing incidence of interference by the tobacco industry’. Notifying states and union territories that have not established empowered committees (Himachal Pradesh, Maharashtra and Jammu and Kashmir) have also omitted measures to limit interactions or reject non-binding agreements, with the tobacco industry. While district level notifications in West Bengal omit such a committee, both Darjeeling and Howrah require officials to ‘limit interactions with the tobacco industry’ and reject corporate social responsibility (CSR) initiatives.

While two-thirds of notifications specify limiting interactions with the tobacco industry, it is notable that state and district governments have excluded several WHO guideline recommendations. Even the most comprehensive notifications (Mizoram, Tamil Nadu, Karnataka, Kerala, Uttar Pradesh and Meghalaya) directly address only four of the eight WHO recommendations. Moreover, recommendations that require transparency for information provided by the industry, avoid giving preferential treatment and treat state-owned tobacco industry in the same way as any other tobacco industry were excluded across *all* notifications. Only six states (Mizoram, Tamil Nadu, Karnataka, Kerala, Uttar Pradesh, and Meghalaya) have formalised a code of conduct to avoid conflicts of interest for government officials, while explicit measures to regulate government engagement with CSR funding from the tobacco industry are restricted to Darjeeling and Howrah (although Maharashtra and Himachal Pradesh notifications do advise rejection of in-kind sponsorship and funding from the tobacco industry).

### Dynamics of notification

The interview data suggest that the Punjab notification was an important source of ‘lesson drawing’^29^ for other states which subsequently translated and adapted this model. Such policy transfer was facilitated through discussions between Punjab officials and those developing protocols in other states. According to the following Mizoram interviewees, for example, such interactions were pivotal in the formulation of other notifications:

We came up with [the notification] in consultation with ministry officials and Punjab officials then we have modified […] So we came with this notification and contact the other lead[s] and kind of the same process was followed.

We asked Punjab [officials] who had done this before us, and we looked at the FCTC and then we looked at what Punjab had done and from there developed our protocol.

The substantive content of the Punjab notification performed an important legitimating function by establishing a legal precedent for subnational policy action. This, in turn, has translated into notifications drawing on precedents as a source of validation. Hence, Karnataka’s creation of an empowered committee cites such mechanisms being established by state governments in Punjab, Mizoram, Bihar and Tamil Nadu, and invokes the Maharashtra, Jammu and Kashmir and Himachal Pradesh governments as having instructed all officers not to participate in industry activities and to reject any sponsorship or funding.

Beyond lesson drawing between state governments and district administrations, the interview data highlight the catalytic role of civil society organisations (CSOs) in advancing Article 5.3 notifications, marked by concerted sensitisation efforts and technical support. CSOs have supported policy development by, inter alia, organising high-level workshops to sensitise government officials, assisting state governments in ensuring that Article 5.3 is on the agenda of State Level Coordination Committees, providing technical support to state tobacco control teams and in monitoring and protesting against tobacco industry interference in public health policy. An important aspect of these advocacy efforts is the extent to which the activities of informal but cohesive networks of CSOs were mutually reinforcing in building momentum. As interviewees in Bihar and Karnataka reflected:

Civil society organizations have served legal notice to elected leaders when it is known that they are going to participate in the tobacco industry event or such other thing.

The CSOs were responsible for initial sensitization and […] it was a workshop conducted by [a named CSO], where local organizations and CSOs from Bihar took the lead. Officials from all departments were invited and involved. There was training and discussion regarding Article 5.3 and it was collectively decided by the Department of Health and Family Welfare that a notification should be issued.

The above extract illustrates the key role of civil society which the interview data suggest was pivotal in supporting state governments to tackle industry interference. In Himachal Pradesh, a government official noted that the notification process was supported by a prominent CSO, detailing how their Article 5.3 technical support and toolkit^30^ including model protocols for limiting interactions with industry and templates for an inter-sectoral committee had enabled sensitising stakeholders. One official described how such resources and technical support were made available ‘at the request of state government’.

The significance of this toolkit is suggested by widespread operationalisation of measures to limit interactions with the tobacco industry and to establish empowered committees. This is consistent with accounts provided by government officials, with one interviewee in Punjab describing how, after attending a sensitisation workshop, ‘we knew exactly what should be done.’ A government official in Karnataka highlighted the value of technical inputs ‘that helped us in framing’ the notification in conjunction with district level officials.

This indicates the importance of high levels of engagement and collaboration between civil society and state government officials. One Punjab advocate described CSOs as “playing as a catalyst” to the state government, while recognising that Punjab’s success owed much to having “been very lucky in the state with the health and government officials very proactive and they were sensitized about the issue” of tobacco industry interference. One civil society representative in Bihar explained the state’s notification as “the outcome of strong collaboration between CSOs and government,” emphasing the technical support provided by CSO via “a high-level consultation [that] was organised under the Chairman of the Chief Secretary.”

### Impacts of Article 5.3 notifications on practice

Article 5.3 notifications have incorporated a significant focus on managing the fundamental conflict between tobacco industry and public health interests. Mizoram, Karnataka, Kerala, Uttar Pradesh and Meghalaya have all adopted protocols specifying that government officials ‘shall avoid the creation of any perception of real or potential partnership or cooperation with the tobacco industry’ and requiring that meetings with the tobacco industry ‘shall be conducted only in the event that it is strictly necessary.’^14^ The interview data suggest strong awareness among health officials of Article 5.3 measures, with one Karnataka official describing how health officials had come to view avoiding conflict of interest as an ethical imperative that formed part of their duties:

I mean it’s logical that it’s very clear that there should not be any conflict of interest. So, there is nothing much beyond that and it lays down the norms for reaching out or meeting the tobacco industry. So, it’s all about what are the norms and the protocol to be followed.

The protocols appear to have had some influence on how state officials approach engagement with the tobacco industry, with the data suggesting that implementation had begun to circumscribe some government-industry interaction. A health official in Mizoram stated that “I’m not supposed to interact. So when [an official] called me up, I told her, I cannot - I’m not supposed to meet people from the tobacco industry.” A health official from Bihar similarly noted that measures to limit interaction with the tobacco industry were ‘helpful, as they prevent us from any meeting’. The obligation of government officials to inform empowered committees of interactions with the tobacco industry appears to have reduced industry requests for meetings with health officials. As the following interviewee from Himachal reflects, transparency requirements for meetings approved by empowered committees had operated to limit interactions:

After the constitution of an empowered committee actually no one approached [health officials] to meet. I think that was the impact and that is pretty good as they don’t want to meet us. They know that the agenda will be set and we will be recording the minutes of the meeting. So, nobody actually requests meetings.

The codification of protocols in notifications also appeared to have shaped administrative practices across state health departments. In Punjab, one health official detailed how protocols for necessary interactions were designed to prevent the industry from misrepresenting any interactions:

Before the meeting, it must be made clear that such interaction does not imply partnership. They should not project as if they are partnering with the government. The meeting should be brief, held preferably in a government setting and not in a private building or a hotel and have written set agenda. All the meetings should have the minutes of meeting.

Alongside signs of progress, the data also highlight a concern among interviewees about the capacity of empowered committees to actively regulate interactions with tobacco industry representatives. A Bihar official noted that, while such committees were a key accountability mechanism, “it is only on paper unless a department comes and tells [the committee] that tobacco industry wants to meet.” Indeed, a civil society representative in Punjab notes how government-industry interactions have continued ‘even after having an empowered committee and even after having a code of conduct in everything’, while a legal consultant highlights similar concerns about the scope to hold actors to account:

There is no guideline on what [happens] if the meeting is in violation of the code of conduct. How do you proceed and what action would you take? […] it’s not very clear on the enforcement part. So, in law there seems to be some basic guidelines that *if there is a meeting these are the conditions that need to be followed*, but no clear guidelines as to what happens when a person sees some sort of violation of this code of conduct.

These concerns about the ability of empowered committees to regulate government-industry interaction were linked to the perception among health officials that colleagues in other government departments were largely unaware of and not actively engaged with Article 5.3. This was summarised by a civil society representative, who observed that a priority for improved implementation ‘would be to reach out to different departments and not just health and sensitise officials on Article 5.3 notifications, how to conduct future interactions with the tobacco industry.’ Another health official reiterated this challenge of sensitisation, describing how they had circulated the notification and tried to engage officials in non-health departments calling ‘them once or twice and formally and informally giving them notices. But, really, [there] is a long, long way to go’.

The interview data also demonstrate how implementation of WHO guideline recommendations at state level can be seen as conflicting with national policies. This tension across issue areas and across different levels of government is particularly evident with respect to limited efforts to regulate tobacco industry CSR. The Government of India’s 2013 revision to its Companies Act requires major businesses to allocate at least 2% of net profits in the previous 3 years to CSR activities,^31 32^ which in the case of the tobacco industry is difficult to reconcile with Article 5.3 guidelines. An interviewee from West Bengal noted that one tobacco company had ‘co-sponsored’ nutrition-related programmes in the state, reflecting that ‘according to Article 5.3 we should not be doing that—the government should not be taking those sponsors.’ A civil society representative from Karnataka similarly reflected how mandated CSR activities undermined principles within the state government’s Article 5.3 notification:

[…] as per the Company Act, they (industry) can invest their profits in the CSR activities. However, as mentioned in the 5.3 notification, CSR is a violation of FCTC 5.3. But again, we have to train our officers, we need to sensitize them regarding this issue. If the industry is doing CSR activities hiding their name, their logo or their deeds - that is also a violation.

## Discussion

Building on the initial example of Punjab’s 2015 notification of measures to implement Article 5.3, the spread of state- and district-level policies examined here provides a vivid illustration of the opportunities federal structures can provide for accelerating tobacco control. Their gaps and omissions notwithstanding, the scale and significance of these innovations should not be underestimated. In terms of coverage, these policies apply for governments with a combined population of over 750 million^33^ and so comfortably exceeding those of the 27 member states of the European Union^34^ or the WHO Eastern Mediterranean Region.^35^

While Article 5.3 implementation guidelines recognise the importance of ensuring engagement of public officials across multiple levels,^14^ the academic and policy literatures have largely been silent on the opportunities and challenges posed in tackling industry interference beyond national governments. An FCTC Secretariat report outlining good country practices in Article 5.3 implementation, for example, makes no substantive reference to subnational approaches to managing industry interference.^36^ Given that state and local governments have long provided settings for innovation in politically challenging areas of tobacco control,^2 11^ this risks neglecting a potentially significant means of building momentum in addressing the principal priority for advancing international implementation of the FCTC.^6^

This analysis of the dynamics of Article 5.3 notifications across state and district levels highlights the significance of CSOs in driving FCTC implementation.^10^ The success with which coalitions of actors mobilised to identify and leverage opportunities to innovate across diverse contexts highlights the importance of strategic adaptation and flexibility in contexts where governance functions operate across multiple levels. The importance of ‘venue shopping’, of proactively selecting policy contexts where actors can most effectively advance their preferences, is familiar in studies of corporate political activity^37 38^; this analysis demonstrates its importance to tobacco control in federal or devolved political systems. Policymaker accounts of support provided by civil society actors demonstrate scope for CSOs to contribute to effectively building capacity for tobacco governance; the clear value afforded to workshops, technical resources and legislative support illustrates the importance of continued investment in supporting policy advocacy to advance international tobacco control.

In building on subnational policy developments, the adoption of a national Code of Conduct for Public Officials^39^ in July 2020 has been welcomed as a significant step in promoting compliance with Article 5.3 implementation guidelines and preventing tobacco industry interference in India.^40^ The Code’s provisions to limit and require transparency in interactions with the tobacco industry, to prevent partnerships and voluntary agreements and to manage conflict of interest among officials reflect measures common across most of the state- and district-level notifications examined here. While it offers a more expansive approach to denormalising ‘partnership, collaboration or agreement with the tobacco industry’^39^ and may provide a starting point for further developments at national level, the Code alone cannot provide the additional enforcement that could strengthen tobacco control governance in a multilevel system.

Across the policy initiatives reviewed here, there is a dearth of measures that explicitly seek to denormalise and regulate tobacco industry CSR initiatives. Only the West Bengal districts of Darjeeling and Howrah explicitly address this key measure, a policy gap that reflects tensions between FCTC implementation guidelines and the requirement in India’s Companies Act 2013^41^ that large companies spend at least 2% of average net profits on CSR activities. While a 2016 ‘clarification’ by the Ministry of Corporate Affairs purported to reconcile this requirement with COTPA’s prohibition of direct and indirect forms of tobacco advertising, promotion and sponsorship,^42^ analyses of tobacco industry interference in India have highlighted the ongoing strategic significance of CSR.^24 32^ Another key omission in state-level notifications is the absence of provisions to prevent giving preferential treatment to the tobacco industry or treating state-owned interests differently to the wider tobacco industry. Such silence is perhaps to be expected given complex national politics and uncertainty regarding regulatory authority at subnational level. There have been regular reports of India’s tobacco industry continuing to benefit from measures including tax exemptions for bidi manufacturers and subsidies for tobacco growers and exporters.^24 43^ Similarly, the absence of reference to regulating tobacco industry interests is significant given the complex structure of investments by state-run insurance companies and other public bodies in the tobacco industry.^44^

In one core respect, the national Code of Conduct fails to keep step with the progress made by most state- and district-level initiatives. With the exception of the union territory of Jammu and Kashmir, the policies reviewed here incorporate clear recognition that a whole-of-government approach is necessary to minimise tobacco industry interference. In most notifying states this needs to be addressed via the establishment of a multisectoral empowered committee, specifying the involvement of diverse government departments and agencies; the Government of Maharashtra did not provide for such a committee but clearly envisage broad engagement in Article 5.3 implementation efforts, with restrictions applying to ‘all government departments in the state and their offices/agencies’.^14^ While the title of the national policy identifies it as a ‘Code of Conduct for Public Officials’, its application is restricted to ‘Officials of Ministry of Health and Family Welfare, its Departments and all the autonomous institutions and Offices under its jurisdiction and to any person acting on their behalf’.^39^ This narrowing of scope is inconsistent with Article 5.3 guidelines and reinforces arguably the most important barrier to their effective implementation, namely the sense that responsibility to minimise industry interference is restricted to health officials that is evident in interview data here and recognised internationally in available literature.^4 45 46^ The gaps in existing provision highlight the need for a more expansive national policy that could build on the profusion of state- and district-level initiatives to consolidate effective multilevel governance for tobacco control. While recent reports monitoring tobacco industry interference in India indicate some initial grounds for cautious optimism,^23–25^ limited progress has been made towards comprehensive protection, particularly given that the majority of states have not adopted notifications to implement Article 5.3 measures. The process of revising COTPA provides an important opportunity to consolidate those initiatives undertaken by the states and districts analysed here and to accelerate and underpin tobacco control in India.^47^

**What this paper adds**

- Opportunities to advance the WHO Framework Convention on Tobacco Control implementation at subnational level have received limited attention. With a federal political system and a population of 1.38 billion, India represents a key case study for such analysis. This study examines how Article 5.3 guideline recommendations have been operationalised by India’s state and union territories.
- Two-thirds of Article 5.3 notifications specify limiting government-industry interactions, but other WHO guideline recommendations are notable omissions.
- The results suggest the importance of ‘lesson drawing’ and the catalytic role of civil society in sensitising state and district governments, while measures to limit interactions appear to have influenced how state officials engage with the tobacco industry.
- Our study highlights the significant potential of state and district level policy making as settings for policy innovation in tobacco control governance.

## Data Availability

No data are available.
